# DuchenneConnect Registry Report

**DOI:** 10.1371/currents.RRN1309

**Published:** 2012-03-13

**Authors:** Vanessa Rangel, Ann S. Martin, Holly L. Peay

**Affiliations:** ^*^VP Genetic Services, PatientCrossroads; ^†^DuchenneConnect Coordinator, Parent Project Muscular Dystrophy and ^‡^Senior Director, Parent Project Muscular Dystrophy

## Abstract

Research activity in Duchenne/Becker muscular dystrophy has surged in recent years, requiring robust information networks to support ongoing development. Established by Parent Project Muscular Dystrophy in late 2007, DuchenneConnect was created to bridge the information gap between care providers, researchers and the patient community, thereby addressing medical care needs and accelerating the pace of therapeutic advancements. This report represents the first in a new series that will be regularly shared by DuchenneConnect and PPMD. Data in this report was collected through June 2011.

## Background

Research activity in Duchenne and Becker muscular dystrophy (DBMD) has surged in recent years, requiring robust information networks to support therapeutic development. As of November 2011, Clinicaltrials.gov lists more than 10 active clinical trials in DBMD (www.clinicaltrials.gov). In addition, the publication in 2010 of care recommendations for Duchenne ([1] [2]) marked a watershed moment for families affected by and clinicians caring for individuals with DBMD.

This recent progress makes close communication between stakeholders in the DBMD community even more vital, e.g., to disseminate and educate about care recommendations, provide feasibility data to researchers and industry, collect natural history data, and facilitate recruitment into research studies. Established by Parent Project Muscular Dystrophy in late 2007, DuchenneConnect was created to bridge the information gap between care providers, researchers and the patient community, thereby addressing medical care needs and accelerating the pace of therapeutic advancements. 

## The Registry Model

The registry model (as described by PatientCrossroads, www.patientcrossroads.com) describes an ideal registry process; this model is consistent with and in part informed by the DuchenneConnect registry. 

***Foundations or institutions*** sponsor registries, acting as the trusted gatekeeper.

***Patients*** register and provide detailed information about their health history.

***Coordinators*** curate patient data, answer questions, and assist with participation. 

***Clinicians/Researchers*** have access to aggregated, de-identified patient data which serves as pilot data, to develop research ideas, and to answer specific research questions.

***Industry*** uses the de-identified patient data for study planning and pre-screening for enrollment. 

## DuchenneConnect Registry

DuchenneConnect is a patient-report registry and educational resource for individuals with Duchenne and Becker muscular dystrophy, carrier females, and families of affected individuals.  The registry can be accessed at www.duchenneconnect.org.  Clinicians and researchers have been informed about the registry through presentations at professional and advocacy conferences, targeted communications, and online outreach efforts. Affected individuals and families are often informed about the registry by clinicians or learn about it through advocacy organizations. 

Registry data is entered by parents/guardians of affected individuals, individuals with DBMD, and rarely by healthcare providers.  Each participant’s data is accessed through a unique ID and password, which maintains security and allows participants to update their data. Participant data is curated by the DuchenneConnect Coordinator and we request updates to participant profiles every 6-12 months.  Data are maintained in a HIPAA-compliant database.  This article provides descriptive data collected from the DuchenneConnect registry between November 2007 and June 2011.  The DuchenneConnect registry data is exempt from IRB review as existing data that is publically available in a de-identified format.  

## Uses of DuchenneConnect 

DuchenneConnect is used to educate families about research, inform about natural history, facilitate economic modeling and feasibility planning for industry, and serves as a recruitment tool.  Increasingly, DuchenneConnect is partnering with academic partners to collect additional data to answer specific hypotheses about the health and psychological effects of DBMD.  See Table 1 for more detail about research announcements through DuchenneConnect. 

**Table:** 

**Table 1. Clinical Trial & Research Study Announcements Summary 2010-2011 **			
**Posted to Homepage**		**Targeted Email Announcements**	
Featured Studies & Study Updates	24 studies	Studies for Targeted Notification	11 studies
Average Number of Pageviews	Mean 975 views/study (range 123-3184)	Patients Who Meet Pre-Screen Criteria	1416 patients

Table 1. Announcements by the DuchenneConnect Registry.

In addition to the connection to front-line information about opportunities to participate in research, participants rely on the DuchenneConnect program as a source of expert knowledge about clinical care, genetics, and research participation. Participants have access to the Connect Coordinator (a certified genetic counselor), the registry team, and advisors.  To date, more than 1,000 questions have been asked of the DuchenneConnect team (see Figure 1).  

**Figure:**
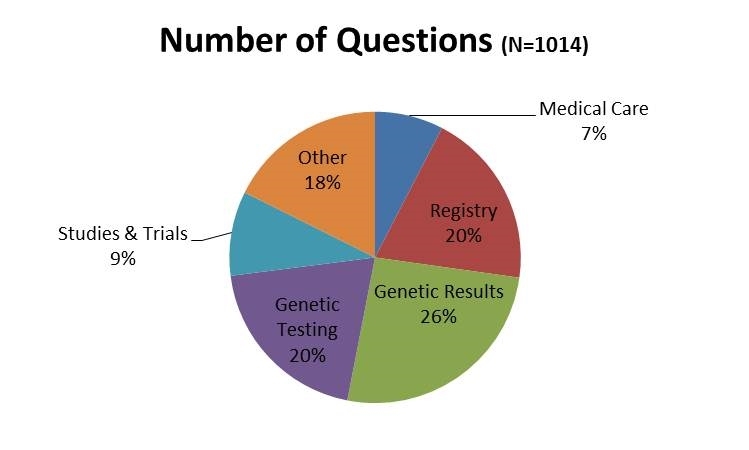
Figure 1. Categories of questions asked to registry staff.

## Summary of Participants 

DuchenneConnect is the largest US-based registry of Duchenne/Becker muscular dystrophy. As of June 2011, the registry represents 1,595 patients with muscular dystrophy, 98 carrier or at-risk females and more than 300 professionals with an interest in muscular dystrophy (see Figure 2).  Of all patient participants, 69% (n= 1213) have submitted a health history survey. Eighty six percent (n=1366) of patient participants report having genetic testing, of which 47% (n= 647) have submitted a copy of their genetic test results.  See Figure 3 for a description of participant characteristics. 

**Figure:**
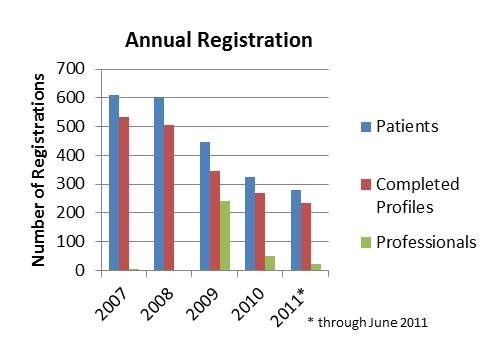
Figure 2. Annual total registrations by year Nov 2007 – June 2011.

**Figure:**
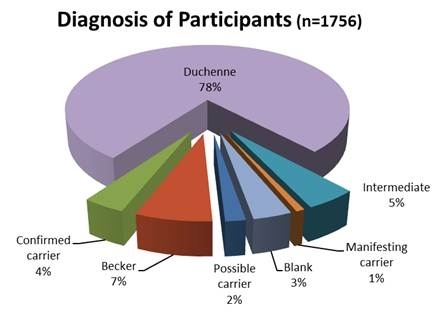
Figure 3. Diagnosis reported by registry participants.  

### Demographics

 The mean age of participants with Duchenne is 15.0 years (range 8mos-64y) and for participants with Becker is 26.6 years (range 1.3y-69y).  See distribution data in Table 2.

**Figure:**
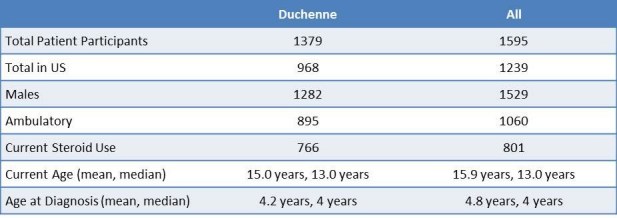
Table 2. Summary of registry participants as of June 2011. Patients include those with Duchenne, Becker, and Intermediate muscular dystrophy.

The majority of participants (77%) chose Caucasian as their racial background.  Though Duchenne/Becker is presumed to be pan-ethnic, participation rates among individuals of African American background are lower than expected, suggesting additional outreach and educational efforts are warranted to further engage this community. 

DuchenneConnect is targeted toward participants in the US.  However, the registry welcomes individuals from any country to participate and has previously offered the survey in Spanish. (This capability will return in the near future, and the survey will also be offered in additional languages.) To date, participants from 78 countries are represented in the registry (see Figure 4). Languages spoken by participants include English, Spanish, German, French, Chinese, Italian, and Vietnamese. 

**Figure:**
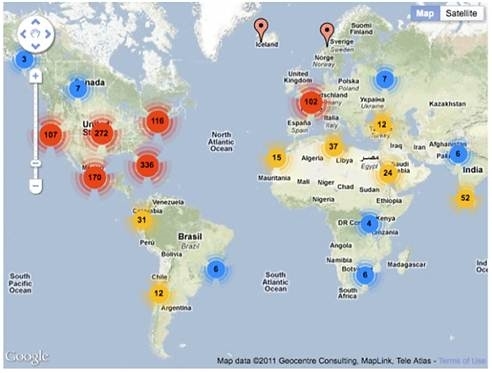
Figure 4. DuchenneConnect continues to draw participants around the globe. The colored circles reflect areas with more/fewer participants.

### Age at Diagnosis 

Frequently, families suspect that something is wrong regarding the health of their child before a diagnosis is established. Among the total group of affected participants, the mean age of diagnosis was reported at 4.8 years.  The mean age at diagnosis for participants with Duchenne was 4.2 years, and for participants with Becker the mean age at diagnosis was 10.8 years.  For Duchenne the symptoms were first recognized on average 1.3 years prior to diagnosis, and for Becker 3.2 years prior to diagnosis. Figure 5 shows age at symptom onset vs. age at diagnosis for the Duchenne/Becker group. 

**Figure:**
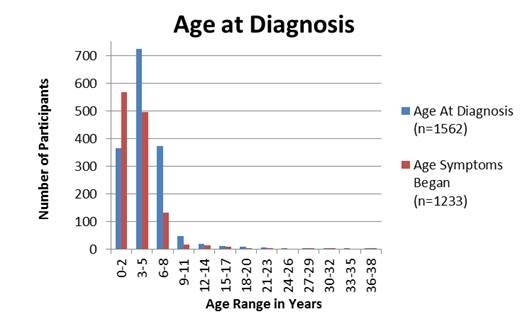
Figure 5. Reported ages of participants at diagnosis (n=1562) and onset of symptoms (n=1233).

### Family History

Dystrophinopathies are X-linked genetic conditions, and in about 70% of cases the mother is expected to be a carrier and about 30% are expected to result from new mutations.  Of 1,680 respondents, 416 report that other family members have a similar muscle disease. Of 351 who provided additional detail, 38% identified a brother(s), 15% identified an uncle(s), 16% report a cousin(s), and 12% identified a mother. (Because of the lower prevalence of manifesting carriers and the fact that carriers are permitted to enter their data in the registry, the majority of identified mothers are expected to represent known carriers vs. manifesting females.) 

### Ambulation

To date, loss of ambulation is an inevitable milestone in the progression of DBMD, though the timing of loss of walking varies considerably between and within Duchenne and Becker muscular dystrophies. Figure 6 shows the status of walking among the patient participants. Of 1,575 participants who responded to this question, 1,060 (67%) are ambulatory and 508 (33%) are non-ambulatory. Of the non-ambulatory group, 1.4% have Becker, 30% have Duchenne, and 1.0% have an intermediary phenotype. 

**Figure:**
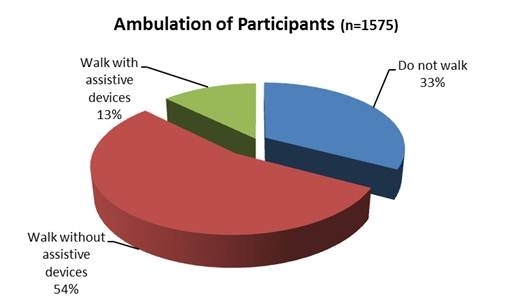
Figure 6. Ambulation among patient participants in the Registry.

The mean age of full-time wheelchair use for those with Duchenne is 10.5 years, and for those with Becker is 21.5 years (see Figure 7).

**Figure:**
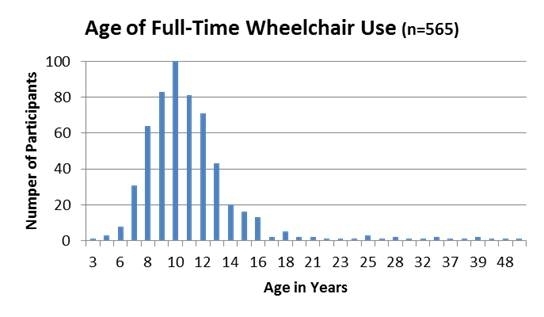
Figure 7. Reported age of full-time wheelchair use.

### Corticosteroid Use 

Use of corticosteroids remains of strong interest to the community, especially following the adoption of the 2010 care recommendations for Duchenne muscular dystrophy that confirm previously published basic guidelines as to their use ([1]).  Of DuchenneConnect participants, 22% currently use prednisone and 29% currently use deflazacort.  Thirty-six percent of respondents have never used corticosteroids. Ten percent of respondents have used corticosteroids in the past but no longer use them. Two percent were unsure about their corticosteroid use.

Among 1,564 patient participants:

      28% (444) have Duchenne and use deflazacort

- 21% (322) have Duchenne and use prednisone
- 1% (12) have Becker and use either deflazacort or prednisone
- 1% (23) have an intermediate presentation and use either deflazacort or prednisone

Of 1,564 participants who provided data about their ambulatory status and their corticosteroid use:

- 40% (624) are ambulatory and use steroids
- 26% (411) are ambulatory but not using steroids
- 11% (176) are not ambulatory and using steroids
- 20% (319) are not ambulatory and not using steroids

Figures 8 and 9 represent corticosteroid use among ambulatory and non-ambulatory patients.

**Figure:**
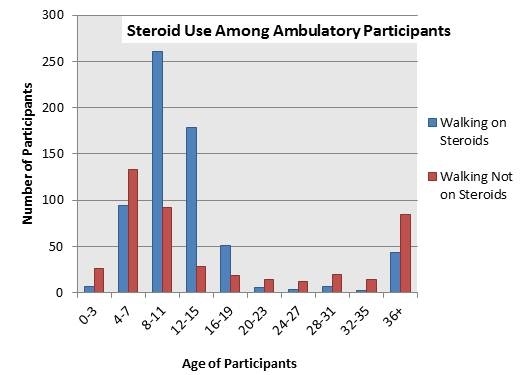
Figure 8. Distribution of ambulatory patients with and without steroids by age.

**Figure:**
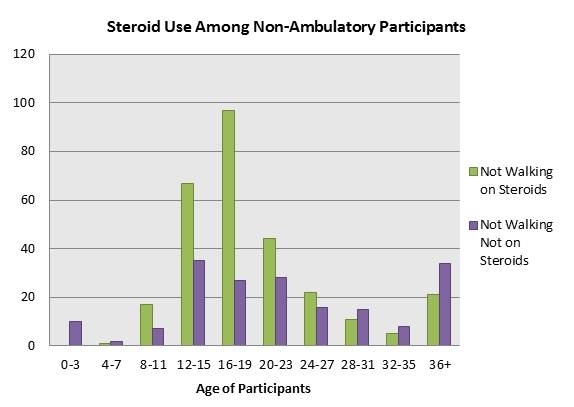
 Figure 9. Distribution of non-ambulatory patients with and without steroids by age.

### Genetic Mutation Status

Dystrophinopathies are caused by a variety of types of mutations in the dystrophin gene (exonic deletions and duplications, a range of smaller mutations within exons, as well as rare deletions to non-coding regions).  Molecular genetic testing for Duchenne/Becker has experienced vast improvements in the past decade, and is currently a recommended aspect of confirming a diagnosis of muscular dystrophy ([1]).  In the registry questionnaire, participants may enter the type and location of the causative mutation. This information is curated by the DuchenneConnect Coordinator.  We encourage all participants to submit a copy of their genetic testing report, which is reviewed by the Coordinators and results are entered into the registry using the standard nomenclature, according to the NCBI Reference Sequence (NM_004006.2).  

Of 1756 participants, 1,432 (82%) indicated they had genetic testing, and 234 (14%) indicate they have not had or were unsure if they have had genetic testing.  Ninety participants (5%) did not indicate whether they had genetic testing. Among 682 who submitted results, the DuchenneConnect Coordinator determined that 34% (232) warrant additional testing to further identify or interpret the results. Of 988 participants who described the mutation, 35% (354) have not submitted a copy of the test report to DuchenneConnect.  

For the Duchenne group, 74% (612) of participants report that results of genetic testing described a deletion one or more exon in size, whereas in Becker 63% (34) report a deletion one or more exon in size. Duplications one or more exon in size are reported by 10% (79) of participants with Duchenne and 11% (6) with Becker. See Table 3 for a summary of mutation status by diagnosis.  Participants who have not had comprehensive genetic testing (i.e., testing able to identify less common types of mutations) may lead to an over-representation of more commonly detectable mutation types. 

**Figure:**
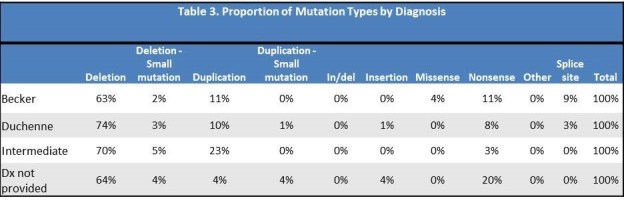
Table 3. Types of gene mutations by diagnosis (n=912). Key: Deletion & Duplication refer to mutations of one or more exons in size typically detected by array, MLPA or similar technologies. All other mutation types refer to smaller mutations typically identified by sequence analysis or similar technologies. In/del refers to co-localized insertion and deletion of base pairs.

### Pulmonary Status

Individuals with DBMD experience progressive deterioration of respiratory muscle strength resulting in complications such as insufficient cough, hypoventilation, and ultimately respiratory failure ([2]), and thus tracking of pulmonary status is a core component to care.  Of 1552 patient participants, 554 (36%) report they have had a forced vital capacity test (a minimum assessment of pulmonary function), 826 (53%) have not had a forced vital capacity test, and 172 (11%) are unsure whether the test has been performed. 

 Of 905 patient participants who responded to this item, 203 (22%) of participants with Duchenne and 7 (1%) of participants with Becker participants report using a breathing device. Additionally, 592 (65%) of those with Duchenne and 59 (7%) Becker participants report not using a device; 39 patients with an intermediate phenotype do not use a breathing devise.  Among devices used, 37% (79) use Cough Assist and 36% (78) use BiPAP. Figure 10 provides additional detail on use of breathing devices.

**Figure:**
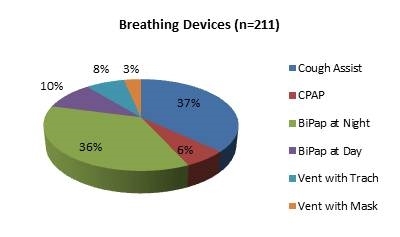
Figure 10. Use of breathing devices reported by participants.

### Cardiac

Progressive cardiomyopathy is a major source of morbidity and mortality in DBMD, and there is disease in the myocardium well before the onset of clinical symptoms ([2]). Of 1565 respondents, 81% (1265) of participants report they do not have cardiomyopathy; this includes 80% (1100) of those with Duchenne, 82% (94) of those with Becker, and 86% (71) of those with an intermediate phenotype. Sixteen percent (216) of those with Duchenne, 15% (17) of those with Becker, and 10% (8) of those with an intermediate phenotype report having cardiomyopathy. The remaining individuals reported not knowing whether they had cardiomyopathy.  Figure 11 shows information on age and diagnosis in those who report having cardiomyopathy.  

**Figure:**
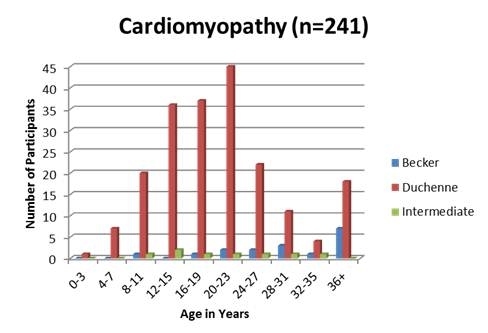
Figure 11. Participants reporting a diagnosis of cardiomyopathy, grouped by ages and diagnosis.

 Of 1581 patient respondents, 1,005 (60%) report not taking any cardiac medications. Seven percent (122) take CoEnzyme Q10, 8% (140) take Lisinopril, and 6% (110) take Enalapril. Use of other cardiac medications is reported among less than 5% of participants 

### Supportive Therapies

The DuchenneConnect registry collects data regarding participants’ use of many supportive therapies, including health therapies, rehabilitative therapies, vitamins/supplements/other medications, and alternative/naturopathic therapies. Physical and occupational therapies are among those most frequently used by participants (34% and 17%, respectively).  See figure 12.

**Figure:**
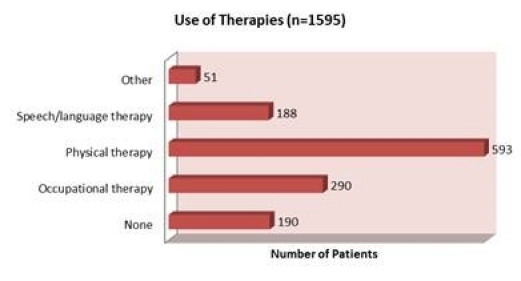
Figure 12.  Most frequently used therapies reported by participants.

 Calcium, Coenzyme Q10, and vitamin D are among the most common supplements used (17%, 13%, 21%, respectively); see table 4. 

**Figure:**
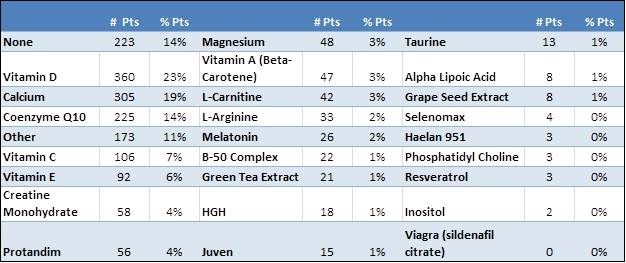
Table 4. Use of supplements, vitamins, other medications reported by participants  (participants can choose more than one).

## Summary

The DuchenneConnect registry holds data about DBMD of interest to academic researchers, clinical investigators, and industry.  This includes family history, genetic mutation status, ambulation, corticosteroid use, pulmonary and cardiac function, and supplement use. De-identified data is made available to academic and industry partners to advance our understanding of natural history and facilitate the drug development and clinical trial process.  Participants in the registry benefit from educational materials and targeted contacts about relevant clinical trials. Looking forward, a new report of selected registry data will be available biannually through the registry website.  Bivariate and multivariate analyses of clinical interest are in process and will be reported in the near future.

## Acknowledgments

The DuchenneConnect team extends its heartfelt gratitude to all the families who have provided information to the registry.  Sharing their knowledge and experience empowers families and enables them to directly impact research and advancements in muscular dystrophy.

## Funding information

DuchenneConnect is supported by Parent Project Muscular Dystrophy. 

## Competing interests

The authors have declared no competing interests.
